# Supplementary material for: Identification of photoperiod-induced specific miRNAs in the adrenal glands of Sunite sheep (Ovis aries)
Source: Front Vet Sci. 2022 Jul 22;9:888207. doi: 10.3389/fvets.2022.888207 (PMC9354845; doi:10.3389/fvets.2022.888207)
Supplement: Supplementary file 1 [file Data_Sheet_1.ZIP › Supplementary file/miRDeep2 file.zip!/miRDeep2 file/Novel_Structure/SPLP42A2/Novel_538.pdf]

Provisional ID	:	NC_019474.2_31766
Score total	:	2.4
Score for star read(s)	:	-1.3
Score for read counts	:	0
Score for mfe	:	2.1
Score for randfold	:	1.6
Score for cons. seed	:	
Total read count	:	21
Mature read count	:	21
Loop read count	:	0
Star read count	:	0

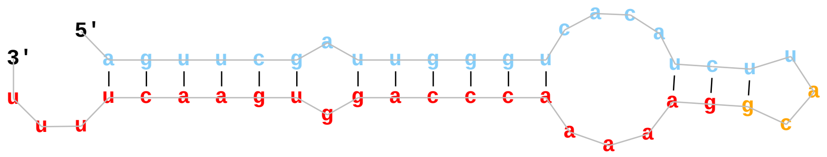

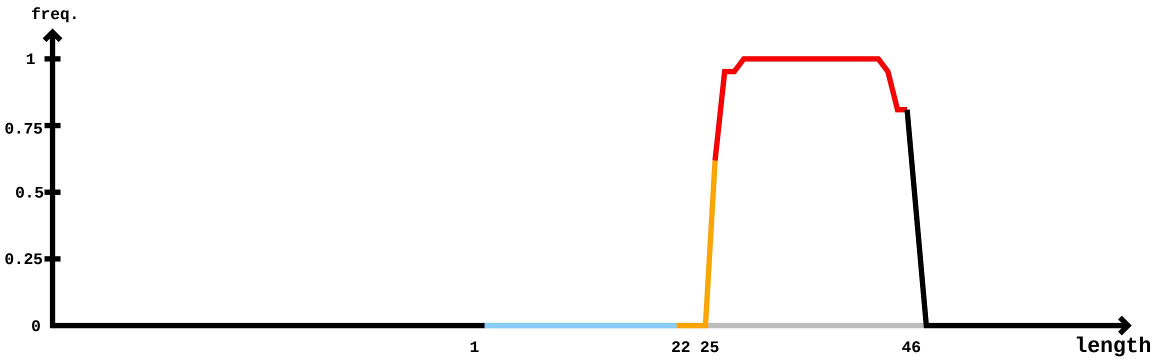

Star Mature

```
5'- cgcguggacuccagccuggggauaacuguuggguuggccaaaaaguucgauugggucacaucuuacggaaaaacccaggugaacuuuuuuggccaacucaacaauacaca  -3'   exp
    ...((...(((((...)))))...))(((((((((((((((((((((.((((((....(((...)))...)))))).))))).))))))))))))))))).......   reads     mm        sample
    ...................................................................gaaaaacccagAugaacuu........................   3         1         seq
    ...................................................................gaaaaacccagAugaacuuuu......................   10        1         seq
    ....................................................................aaaaacccagAugaacu.........................   1         1         seq
    ....................................................................aaaaacccagAugaacuuuu......................   5         1         seq
    ....................................................................aaaaacccaAgugaacuuuuu.....................   1         1         seq
    ......................................................................aaacccagAugaacuuuuu.....................   1         1         seq
```
